# Supplementary material for: Five-year-olds’ facial mimicry following social ostracism is modulated by attachment security
Source: PLoS One. 2020 Dec 29;15(12):e0240680. doi: 10.1371/journal.pone.0240680 (PMC7771852; doi:10.1371/journal.pone.0240680)
Supplement: S1 Table — (DOCX) [file pone.0240680.s001.docx]

| **S1 Table**. Summary statistics of muscle activation: descriptives and correlation analyses | | | | | | | | | | | |
| --- | --- | --- | --- | --- | --- | --- | --- | --- | --- | --- | --- |
|  |  |  |  |  |  |  |  |  |  |  |  |
|  |  |  | *M* (*SD*) | 1. | 2. | 3. | 4. | 5. | 6. | 7. | 8. |
| Includer | Happy | 1. ZM | .26 (.59) | - | -.053 | .503 | .104 | .142 | .665 | .**371*** | -.286 |
|  |  | 2. CS | .14 (.57) |  | - | -.052 | .050 | .132 | .216 | .189 | .188 |
|  | Sad | 3. ZM | .16 (.81) |  |  | - | -.092 | .158 | .123 | **.457**** | .000 |
|  |  | 4. CS | .36 (.72) |  |  |  | - | -.146 | **.473**** | -.169 | .010 |
| Excluder | Happy | 5. ZM | .26 (.68) |  |  |  |  | - | -.115 | **.346*** | -.199 |
|  |  | 6. CS | -.15 (.66) |  |  |  |  |  | - | -.065 | **.358*** |
|  | Sad | 7. ZM | .31 (.82) |  |  |  |  |  |  | - | .054 |
|  |  | 8. CS | -.10 (.77) |  |  |  |  |  |  |  | - |
| *Note. M* = mean, *SD* = standard deviation, * *p* < .05, ** *p* < .01. | | | | | | |  |  |  |  |  |
