# Supplementary material for: Five-year-olds’ facial mimicry following social ostracism is modulated by attachment security
Source: PLoS One. 2020 Dec 29;15(12):e0240680. doi: 10.1371/journal.pone.0240680 (PMC7771852; doi:10.1371/journal.pone.0240680)
Supplement: Attachment — Submitted filename: Response to Reviewers_R1.docx [file pone.0240680.s003.docx]

**PONE-D-20-07094R1: Five-Year-Olds’ Facial Mimicry Following Social Ostracism is Modulated by Attachment Security**

**Reviewer2**

Methods

6) I am not sure what the issue is, as the authors do not mention the test that power was computed for, but even for a one-sided test of a .40 correlation at alpha = .05 a sample of n=59 is needed to achieve 95% power (power at n=36 is approximately .82), and the analyses that are actually conducted are more complex.

**Response:** The sample size was determined based on prior work using a similar approach in which a medium effect size (R^2^ = .39) of attachment on facial mimicry was found (Vacaru et al., 2019). Additionally, a power analysis was performed using a linear multiple regression from the t-test family, two-tailed, 3 predictors (i.e. resistant attachment, avoidant attachment, and their interaction) assuming a medium effect size of .39, with .95 power and α of 0.05. This calculation showed a required sample of 36 participants. We added this explanation to the manuscript (see page 7, lines 163-168).

15) The use of dependent variables is inconsistent across analyses. This can be defended, but please explain why choices were made and add those to the paper.

**Response:** In the first analysis, we investigated whether children display mimicry responses at all, and hence we employed a repeated measure ANOVA with emotion (happy, sad), muscle (CS, ZM) and peer (includer, excluder) as independent variables and mean standardized muscle activations as the dependent variables. This analysis provides information as to whether muscle activation differences exist as a function of the observed emotional expression, displayed by one or the other peer.

In the second analysis, however, our research question was different. We investigated the effect of attachment on the magnitude of the emotional facial response to each peer. To this end, we collapsed the two different muscles into one difference score as an index of a facial configuration: i.e. happy or sad. This choice is also in line with previous work, which first describes an ANOVA approach to investigate muscle activation as a function of the observed emotion, and then creates composite scores for each emotion based on the differences of the selected muscles (Deschamps et al., 2012, 2014; Vacaru et al., 2019). To clarify this point in the manuscript, we added this explanation to the *Statistical Analyses* section (see page 14, lines 329-338).

22) The authors have added a discussion of limitations to the Discussion section. However, there is one limitation that I think requires some more attention. The authors state that parents were present while children played the cyberball game and that parents’ presence may have affected children’s coping with social exclusion. The authors would do well to consider potential differential effects of the presence of parents on securely and insecurely attached children. Also, the parents’ presence should be mentioned in the Method section (e.g. under procedures).

**Response:** We thank the reviewer for this suggestion, which we have now given more attention in the discussion. We agree that the parent’s presence in the room may have had a different effect on securely and insecurely attached children. That is, for securely attached children their parents’ presence may constitute a safe haven (Powell et al., 2009) where children find refuge in a moment of distress (i.e. ostracism in the game). However, for insecurely attached children, the presence of their parents in the room may not instill more safety feelings in the children compared to if they were alone, and hence this may not have alleviated the effects of the stressor. Accordingly, in our study, securely attached children reacted to the excluder peer, possibly also because they felt protected by their parents’ presence in this ostracizing situation. The lack of a response in the insecurely attached children, in contrast, may indicate that they did not feel safe enough to react to the unpleasant situation. We added this explanation in the manuscript (see page 20, lines 476-474).

As a minor comment: The authors refer to parents as parents of babies. They might change ‘babies’ to ‘children’.

**Response:** Thank you for noticing this. We have now replaced ‘babies’ with ‘children’	throughout the manuscript.
